# Financial incentives for smoking cessation among (expectant) parents: a systematic review of facilitators and barriers to implementation

**DOI:** 10.1136/tc-2024-059198

**Published:** 2025-04-22

**Authors:** Linda van der Spek, Leonieke J Breunis, Tessa Scheffers-van Schayck, Linda Bauld, Erwin Ista, Jasper V Been

**Affiliations:** 1 Division of Neonatology, Department of Neonatal and Paediatric Intensive Care, Erasmus MC Sophia Children's Hospital, University Medical Centre Rotterdam, Rotterdam, Netherlands; 2 Department of Epidemiology, Trimbos Institute, Utrecht, Netherlands; 3 Department of Obstetrics and Gynaecology, Erasmus MC Sophia Children’s Hospital, University Medical Centre Rotterdam, Rotterdam, Netherlands; 4 Department of Paediatrics, Erasmus MC Sophia Children’s Hospital, University Medical Centre Rotterdam, Rotterdam, Netherlands; 5 Department of Youth, Trimbos Institute, Utrecht, Netherlands; 6 Usher Institute and Behavioural Research UK, The University of Edinburgh, Edinburgh, UK; 7 Division of Paediatric Intensive Care, Department of Neonatal and Paediatric Intensive Care, Erasmus MC Sophia Children’s Hospital, University Medical Centre Rotterdam, Rotterdam, Netherlands; 8 Section Nursing Science, Department of Internal Medicine, Erasmus MC, University Medical Centre Rotterdam, Rotterdam, Netherlands; 9 Department of Public Health, Erasmus MC, University Medical Centre Rotterdam, Rotterdam, Netherlands

**Keywords:** Addiction, Cessation, Economics, Socioeconomic status

## Abstract

**Objective:**

Financial incentives, provided following validated smoking cessation, hold substantial potential to cost-effectively promote cessation. To facilitate wider adoption, we systematically reviewed evidence addressing the barriers and facilitators to successful implementation of incentive-based smoking cessation interventions among (expectant) parents.

**Data sources:**

We conducted a systematic search to identify scientific and grey literature across nine electronic databases, from inception to 15 August 2024. Search terms included combinations of “smoking cessation”, “incentive”, “pregnancy”, “preconception” and “parent”.

**Study selection:**

Eligible records reported and reflected on the implementation of smoking cessation programmes with incentives for (expectant) parents. Inclusion criteria were applied by two reviewers independently, with discrepancies resolved through consensus. Of 1100 unique records identified, 37 met inclusion criteria.

**Data extraction:**

Characteristics of the studies, interventions, incentives and implementation, along with barriers and facilitators, were independently extracted by two reviewers. Thematic analysis identified barriers and facilitators. Subgroup analysis explored patterns specific to lower socioeconomic populations.

**Data synthesis:**

Studies reported on implementation in the USA (n=18), UK (n=10), Australia (n=4), the Netherlands (n=2), New Zealand (n=1), France (n=1) and international contexts (n=1). Barriers included misalignment with participants’ context and resources, recruitment and retention challenges, limited reliability of abstinence verification and high resource demands. Facilitators included ensuring acceptability, accessibility, feasibility, funding and integration into health services.

**Conclusions:**

With the cost-effectiveness of financial incentives for smoking cessation among (expectant) parents already well-documented, this first systematic synthesis of the barriers and facilitators to implementing them in daily practice offers valuable guidance for advancing implementation efforts.

**PROSPERO registration number:**

2023:CRD42023407648.

**WHAT IS ALREADY KNOWN ON THIS TOPIC**

- Financial incentives can cost-effectively support smoking cessation, including among pregnant women, but implementation among (expectant) parents remains limited.

**WHAT THIS STUDY ADDS**

- This study identifies key facilitators and barriers to implementing financial incentives, offering insights for broader adoption.

**HOW THIS STUDY MIGHT AFFECT RESEARCH, PRACTICE OR POLICY**

- Findings can guide policymakers and professionals to optimise implementation of incentives by addressing contextual fit, integration into care and challenges in recruitment, retention and abstinence verification.

## Introduction

 The detrimental effects of exposure to parental smoking during the periconception phase, pregnancy and childhood have long been acknowledged.^1–3^ Nonetheless, the prevalence of smoking during pregnancy ranges from 8% to 24% in the European Region, the USA and Australia.^4^ Exposure to secondhand tobacco smoke is generally highest among children.^5–7^ Globally in 2017, over 60 000 children under the age of 10 were estimated to have died due to secondhand smoke exposure.^8^ Evidence-based interventions supporting smoking cessation still have limited reach and effectiveness, especially among socioeconomically disadvantaged groups.^9 10^ A meta-analysis indicated that 87% of pregnant women who received a smoking cessation intervention still smoked at the end of pregnancy.^11^ Of those who quit, 43% relapsed within 6 months postpartum.^11^

The provision of financial incentives following validated smoking cessation holds substantial potential to cost-effectively promote and sustain smoking cessation, including among pregnant women.^12–14^ High certainty evidence from meta-analyses showed that pregnant women are twice as likely to achieve sustained abstinence when receiving financial incentives instead of usual care.^13 15^ Financial incentives can be defined as cash or cash-like rewards contingent on the performance of the healthy behaviour targeted by the intervention.^16^ Incentives are known to improve participation rates, sustain engagement and ultimately enhance smoking cessation outcomes.^12^ Therefore, England is now implementing financial incentives for pregnant women nationwide, following National Institute for Health and Care Excellence (NICE) guidelines.^17 18^

Despite the compelling evidence for financial incentives to enhance the adoption and effectiveness of smoking cessation programmes among pregnant women, implementation endeavours in most countries have remained limited.^19–21^ To facilitate wider adoption, a comprehensive understanding is needed regarding the optimal implementation of incentive-based smoking cessation programmes.^22^ Implementation is often impeded by barriers regarding, for example, ethical concerns,^23^ negative public opinion,^24–26^ lack of information,^27^ funding^12 28^ and (remote) biochemical validation.^12 29^ Also, scant attention has been directed towards incentives for parents outside the pregnancy context, while the negative consequences of exposure to tobacco smoke for children extend from the preconception period until beyond pregnancy.^2 30^ Distinct implementation strategies thus warrant exploration, recognising the all-encompassing urgency as well as the differing characteristics, needs and healthcare utilisation patterns among pregnant women, their partners, people with a wish to conceive and parents of children aged 0–18 years, collectively termed here as ‘(expectant) parents’.

To inform future implementation efforts, this study thus aims to systematically synthesise evidence addressing the barriers and facilitators to successful implementation of incentive-based smoking cessation interventions among (expectant) parents.

## Methods

This review is reported according to the Preferred Reporting Items for Systematic Reviews and Meta-Analyses guidelines.^31 32^ The review protocol was registered with PROSPERO prior to data extraction (Registration no. 2023:CRD42023407648, see online supplemental file 1, Protocol). There were no substantive deviations from the protocol.

### Search strategy

A systematic literature search was conducted in eight electronic databases: Embase, MEDLINE (Ovid), Cochrane Central, Web of Science, Google Scholar, PsycInfo, Cumulated Index to Nursing and Allied Health Literature and EconLit, from inception to 15 August 2024. The search strategies were developed in consultation with a medical information specialist. Search terms were customised to each database and included variations of “smoking cessation”, “incentive”, “pregnancy”, “preconception”, “parent” (see online supplemental file 2, Search strategy). Grey literature (eg, opinion papers, guidelines, policy papers and dissertations, produced on any level of government, academics, business and industry, as defined by the Luxembourg definition^33^) was retrieved using the Google search engine, also from inception to 15 August 2024. Additionally, papers citing or cited by papers or topic-related literature reviews were screened using Scopus, following Bramer.^34^

### Study selection

EndNote V.20 was used to manage and screen search results. After removing duplicates, two reviewers (LJB and LvdS) independently screened the identified records for eligibility, first based on records’ title and abstract and subsequently based on full texts. Records reflecting on the implementation of interventions with financial incentives for smoking cessation targeting (expectant) parents were included. The key concept of implementation was defined as the constellation of processes aimed at getting an intervention into use within an organisation or setting.^35 36^ Financial incentives were considered cash and cash-like rewards, including (vouchers for) goods and services, contingent on smoking cessation or abstinence, following the definition by Adams and colleagues.^37^

The current review has a highly comprehensive approach by including studies with any design, as well as a broad range of publication types, in order to identify every relevant record and achieve in-depth insight into the complexity of implementation and context.^38 39^ Primary research reports, editorials, commentaries and book sections were included, whereas conference abstracts, letters, news releases, reviews and study protocols were excluded. Grey literature was also considered for inclusion as recommended in the Cochrane Handbook of Systematic Reviews, offering more in-depth and less selective insight into interventions, their context and implementation.^40–43^ No restriction was imposed on language, publication date or methodological quality.

### Data extraction and quality assessment

Data were extracted from the included records by one reviewer (LvdS) using a customised data extraction form developed for this purpose and collected in tabular form in Excel. The extracted information covered: study characteristics, details of the smoking cessation programme and incentives, intervention effectiveness, facilitators and barriers to implementation, as well as the costs and sustainability of implementation. Incentive interventions were described using the template for intervention description and replication (TIDieR) checklist,^44^ and incentives were described using the framework of Adams and colleagues.^37^ The total sum of incentives per intervention was converted to US dollars with equivalent purchasing power in the study’s publication year, if originally provided in a different currency.^45^ We categorised intervention effectiveness, based on reported effects at the latest data collection point, as either ‘effective’ (statistically significant improvement compared with the control group), ‘promising’ (improvement but no control group or lack of power) or ‘unknown’ (no improvement reported). The quality of included studies was assessed using The Mixed Methods Appraisal Tool^46^ (MMAT version 2018), as recommended for process-oriented reviewing of public health interventions.^47^ In line with the recommendations of the MMAT developers, we present details alongside quality scores, considering studies that meet at least 60% of MMAT criteria as having good methodological quality.^46 47^ The MMAT criterion on complete outcome data was met when primary outcome data was available at the latest collection point from at least 60% of participants. A second reviewer (LJB) independently conducted the quality assessment and extracted data on implementation barriers and facilitators for all records. For other data (ie, study and intervention characteristics), the second reviewer cross-checked 20%^48–57^ of the data extracted by the first reviewer, leading to disagreement or additions on 7% of the items. Disagreements were resolved by discussion, and if needed, by consulting a third researcher (JB).

### Data synthesis

This review followed a convergent integrated approach for mixed methods systematic reviews, meaning that qualitative and quantitative data were synthesised jointly.^58^ Barriers and facilitators were organised and interpreted following the constructs of the Consolidated Framework for Implementation Research (CFIR), comprising the individual, intervention, inner setting, outer setting and implementation process.^59^ Although our protocol specified a deductive thematic synthesis, we adopted a mixed deductive–inductive approach. This meant that barriers and facilitators were first identified inductively, and then deductively matched to the corresponding CFIR constructs. This modification reduced the risk of missing emergent themes that might not immediately align with the constructs in the deductive framework. Next, these barriers and facilitators to the implementation of interventions with financial incentives for smoking cessation among (expectant) parents were presented in narrative and tabular form. Subgroup analysis discerned unique barriers and facilitators to implementation in populations with a lower socioeconomic status (SES). Populations with a lower SES were characterised by the original authors using indicators such as (predominantly) low-income, lower education levels, unemployment, reliance on social security programmes (eg, the US Special Supplemental Nutrition Program for Women, Infants and Children or Medicaid-enrolled) and descriptors like ‘disadvantaged’ or ‘deprived’, applied at either area or individual level.

## Results

### Study selection

**Figure 1:**
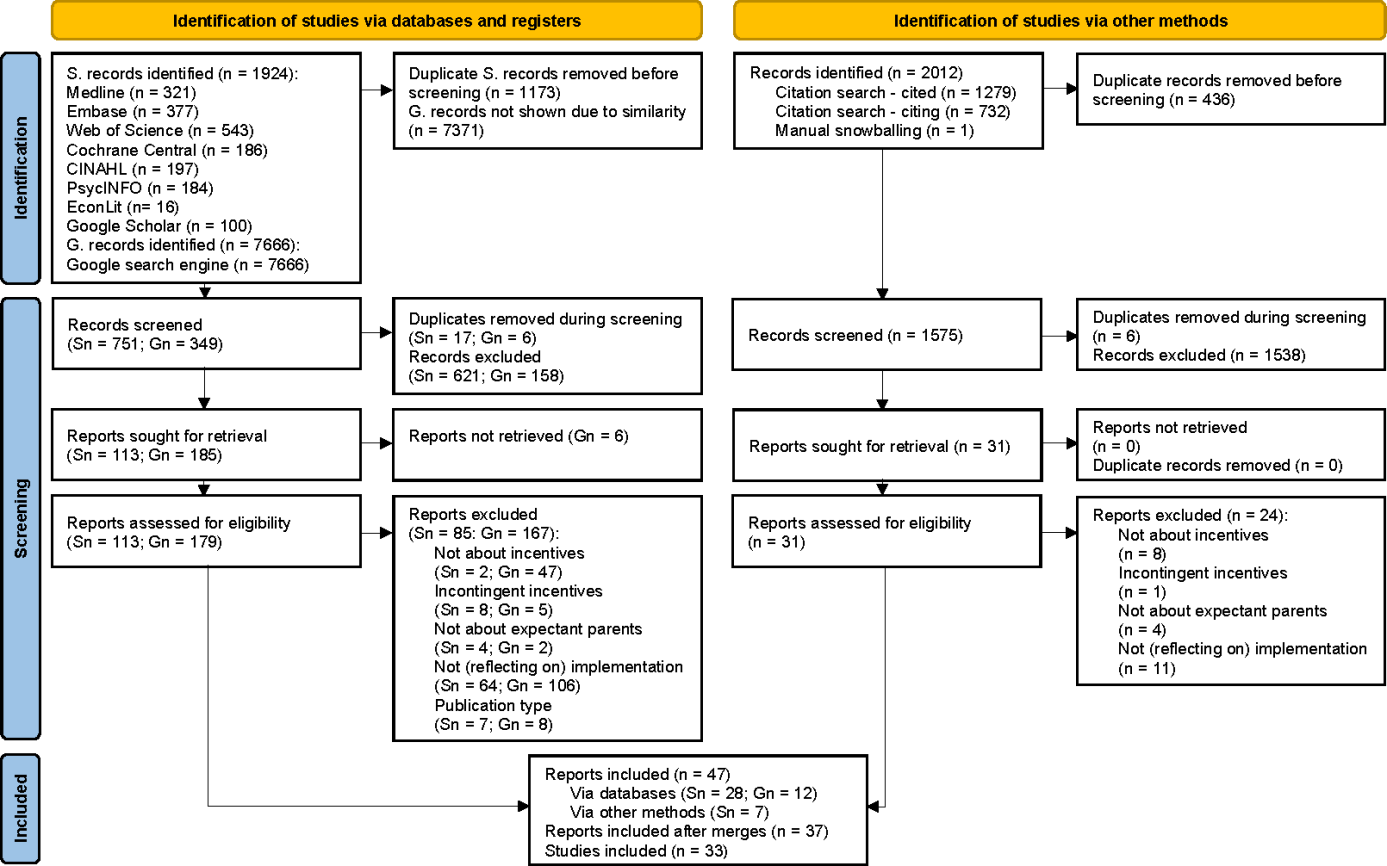
Preferred Reporting Items for Systematic Reviews and Meta-Analyses flow diagram of study selection.^31^ G, grey literature; S, scientific literature.

The literature search yielded 751 unique scientific and 349 unique grey literature records (figure 1). After screening and selection, 28 articles from scientific literature and 12 articles from grey literature were included, supplemented by seven scientific literature articles from citation and reference searches. Data extracted from 10 secondary records,^50 57 60–67^ comprising referenced writing such as editorials, could be merged with the original research records reporting on the empirical data of the same studies. Ultimately, 37 records were included, including 27 original research records and 10 secondary records, together reporting on 33 unique interventions.

### Description of the included literature

Table 1 provides an overview of the characteristics of the 37 included records, published between 2000 and 2024. Four records reflected on implementation indirectly: an editorial^68^ linked to a study conducted in France,^69^ the NICE guideline^18^ and a commentary^70^ reflected on implementation in the context of the UK and an Australian discussion paper^56^ reflected on international evidence of implementation. 33 records reflected directly on the implementation of interventions in the USA (n=18), UK (n=8), Australia (n=4), the Netherlands (n=2) and New Zealand (n=1). 18 of these interventions (55%) were characterised by the original authors as targeting populations with lower SES. Pregnant women were the primary target population of all interventions described. Few also recruited partners or other close contacts (n=3), or women in the preconception (n=1) or postpartum phase (n=1). Support to pregnant women was extended into the postpartum period in about half of the interventions (55%). Regarding methodological quality, 16 out of 27 original research articles met at least 60% of the MMAT criteria and were considered good quality (see online supplemental file 3, Risk of bias (MMAT)).

**Table 1:** Study characteristics

Reference	Study characteristics			Sample characteristics		
**First author and year**	**Publication type**	**Design**	**Location**	**Profile**	**SES**	**N**
Alaniz 2019^71^	Original research	RCT	USA	Pregnant	Low	185
Allison 2023^72^	Original research	MM	UK	Pregnant	Low	45
Askew 2019^48^	Original research	MM	AUS	Pregnant+CC	Low	31
Baker 2018^49^	Original research	RCT	USA	Pregnant	Low	1014
Bou-Samra 2021^91^	PowerPoint presentation	–	AUS	Pregnant	Mixed	10
Breunis 2021^68^	Editorial*	–	–	–	–	–
Breunis 2023^51^	Original research	MM	NL	Pregnant+child wish	Mixed	39
Campbell 2015^54^	PowerPoint presentation	RCT	UK	Pregnant	Mixed	612
Donatelle 2000^52^	Original research	RCT	USA	Pregnant+PP	Low	220
Frankland 2019^55^	Webinar	Case study	UK	Pregnant	Low	680
Gadomski 2011^90^	Original research	nRQ	USA	Pregnant	Low	792
Glover 2015^73^	Original research	RCT	NZ	Pregnant	Mixed	24
Gulliver 2004^81^	Original research	RCT	USA	Pregnant+CC	Mixed	20
Gupta 2015^74^	Journal supplement	–	USA	Pregnant	Mixed	60
Harris 2015^79^	Original research	RCT	USA	Pregnant	Low	17
Hefler 2013^56^	Discussion paper*	Roundtable	–	–	–	–
Heil 2008^89^	Original research	RCT	USA	Pregnant	Low	82
Higgins 2014^87^	Original research	RCT	USA	Pregnant	Mixed	130
Ierfino 2015^53^	Original research	nRQ	UK	Pregnant	Low	239
Jackson 2023^75^	Original research	QD	AUS	Pregnant	Mixed	46
Joyce 2021^80^	Original research	RCT	USA	Pregnant+PP	Low	12
JSI 2009^76^	Evaluation report	–	USA	Pregnant	Low	90
Kock 2024^70^	Commentary*	–	–	–	–	–
Kroder 2022^82^	Original research	MM	NL	Pregnant	Low	9
Kurti 2020^77^	Original research	nRQ	USA	Pregnant	Mixed	150
McMeekin 2023^93^	Original research	RCT	UK	Pregnant	Mixed	944
Miller 2017^86^	Original research	nRQ	USA	Pregnant	Mixed	32
NICE 2021 (updated 2023)^18^	Guideline*	–	–	–	–	–
Ondersma 2012^83^	Original research	RCT	USA	Pregnant	Low	110
Passey 2018^88^	Original research	QD	AUS	Pregnant	Mix	22
Radley 2013^19^	Original research	MM	UK	Pregnant	Low	383
Shields 2016^95^	Report	–	UK	Pregnant+CC	Mixed	384
Too 2021^20^	Original research	nRQ	UK	Pregnant	Low	652
Valencia 2020^78^	Original research	nRQ	USA	Pregnant	Mixed	10
Wen 2019^84^	Original research	nRQ	USA	Pregnant	Low	30
Yon 2022^85^	Original research	QD	USA	Pregnant	Mixed	20
Zhang 2017^92^	Original research	nRQ	USA	Pregnant	Low	869

* Reflected on implementation indirectly.

AUS, Australia; CC, close contact; JSI, JSI Research & Training Institute, Inc; MM, mixed-methods; NL, the Netherlands; nRQ, non-randomised quantitative; NZ, New Zealand; PP, postpartum; QD, quantitative descriptive; RCT, randomised controlled trial; SES, socioeconomic status, either characterised by original authors as lower or mixed/unspecified.

### Description of the interventions

Table 2 gives an overview of the 33 interventions evaluated. Four additional records^18 56 68 70^ were reviewed for their reflections on implementation but did not provide firsthand intervention details and were therefore not included in this table. With regard to promoting smoking cessation, 12 interventions were categorised as ‘effective’ (35%), 9 as ‘unknown or ineffective’ (26%) and 12 as ‘unknown but promising’ (35%), as there was a lack of power or control group. 76% of interventions offered counselling and 39% offered (optional) nicotine replacement therapy. The majority of interventions (82%) was delivered face-to-face, either exclusively or as an addition to other delivery modes. Phone calls,^20 49 71–76^ phone applications,^74 77 78^ websites^51 75 79^ or video calls^80^ were also used for delivery. Two interventions contained a group-based component.^51 81^

**Table 2:** Intervention and incentive details

First author and year	Provider	Delivery	Effectiveness	Incentive	US$	Frequency	Validation
Alaniz 2019^71^	SCC	F2F+phone	Promising	Voucher (R)	140	7	Breath CO, at home
Allison 2023^72^	SCC	F2F+phone	Unknown	Voucher (R)	600	5	Breath CO, at home
Askew 2019^48^	HCP+SW	F2F	Promising	Voucher (R)	130	4	Breath CO, at home
Baker 2018^49^	HCP	F2F+phone	Effective	Voucher (R)	500	2	Breath CO, at home
Bou-Samra 2021^91^	HCP	F2F	Unknown	Voucher (R/S)	?	12	Breath CO, at home
Breunis 2023^51^	RS	F2F+web	Promising	Voucher (R)+service	255	6	Breath CO, on-site
Campbell 2015^54^	SCC	F2F	Effective	Voucher (R)	577	4	Sal-Cot, at home
Donatelle 2000^52^	HCP+RS	F2F	Effective	Voucher (R)	250	10	Sal-Thio, on-site
Frankland 2019^55^	SCC	F2F	Effective	Voucher (R)	209	12	Breath CO, on-site
Gadomski 2011^90^	SCC	F2F	Effective	Voucher (S)	240	12	Breath CO, at home
Glover 2015^73^	RS	Phone+F2F	Promising	Voucher (R)/Goods	135	8	Breath CO, at home
Gulliver 2004^81^	PSY+RS	F2F	Promising	Voucher (R)	?	6	Breath CO, on-site
Gupta 2015^74^	SCC	F2F+phone+app	Effective	Voucher (R)	2400	?	Breath CO, on-site
Harris 2015^79^	RS	Web	Unknown	Voucher (R)+Cash	296	86	Breath CO, at home
Heil 2008^89^	HCP+RS	F2F	Effective	Voucher (R)	1620	36	Breath CO, at home
Higgins 2014^87^	HCP+RS	F2F	Effective	Voucher (R)	1180	36	Breath CO, at home
Ierfino 2015^53^	SCC	F2F	Promising	Voucher (R)	1085	32	Breath CO, on-site
Jackson 2023^75^	RS+SCC	Web+phone	Unknown	Voucher (R)	1476	100	Breath-CO, at home
Joyce 2021^80^	RS	Video call	Unknown	Cash	112	25	Watch+Sal Cot + Ur Cot, at home
JSI 2009^76^	SW+SCC	F2F+phone+postcard	Promising	Voucher (R)	455	11	Breath CO, on-site
Kroder 2022^82^	RS	F2F	Unknown	Voucher (R)	792	4	Ur-Cot, on-site
Kurti 2020^77^	RS+HCP	App	Effective	Cash	1620	40	Breath CO, at home
McMeekin 2023^93^	?	?	Effective	Voucher (R)	538	3	Breath CO
Miller 2017^86^	RS	Web	Promising	Cash	371	84	Breath CO, at home
Ondersma 2012^83^	RS	F2F	Unknown	Voucher (R)	250	5	Ur-Cot, on-site
Passey 2018^88^	HCP	F2F	Promising	Voucher (R)	20	12	Breath CO, on-site
Radley 2013^19^	Ph+SW	F2F	Promising	Voucher (R)	270	15	Breath CO, on-site
Shields 2016^95^	SCC	F2F	Promising	?	116	4	Breath CO, on-site
Too 2021^20^	SCC+Ph	F2F+phone	Effective	Voucher (R)	217	3	Breath CO, on-site
Valencia 2020^78^	RS	F2F+app	Unknown	Voucher (R)	?	28	Breath CO, on-site
Wen 2019^84^	SCC+RS	F2F	Promising	Cash	1025	22	Ur-Cot, on-site
Yon 2022^85^	HCP	F2F	Unknown	Voucher (R)	1115	42	Sal-Cot, on-site
Zhang 2017^92^	HCP	F2F	Effective	Voucher (S)	300	12	Breath CO, on-site

CO, carbon monoxide; F2F, face-to-face; HCP, healthcare professional; Ph, pharmacologist; PSY, (clinical) psychologist; RS, research staff; Sal-Cot, salivary cotinine validation; Sal-Thio, salivary thiocyanate validation; SCC, smoking cessation counsellor; SW, social workers or health and social service professionals; Ur-Cot, urinary cotinine validation; Voucher (R), voucher for a range of goods/services; Voucher (S), voucher for specific goods/services; Watch, smartwatch movement validation.

Each intervention required biochemical validation of abstinence to be eligible for incentives. In 26 interventions (79%), exhaled breath carbon monoxide (CO) was used for biochemical validation. In a few other interventions, urinary cotinine,^80 82–84^ salivary cotinine^54 80 85^ or salivary thiocyanate^52^ were used, or a smartwatch to detect movement associated with smoking.^80^ For over half of the interventions (52%), on-site validation was required, instead of validation at home or another participant-determined site. On average, participants received up to 22 payments, with the total amount averaging a maximum of US$610 per person.

### Barriers and facilitators to implementation

**Figure 2:**
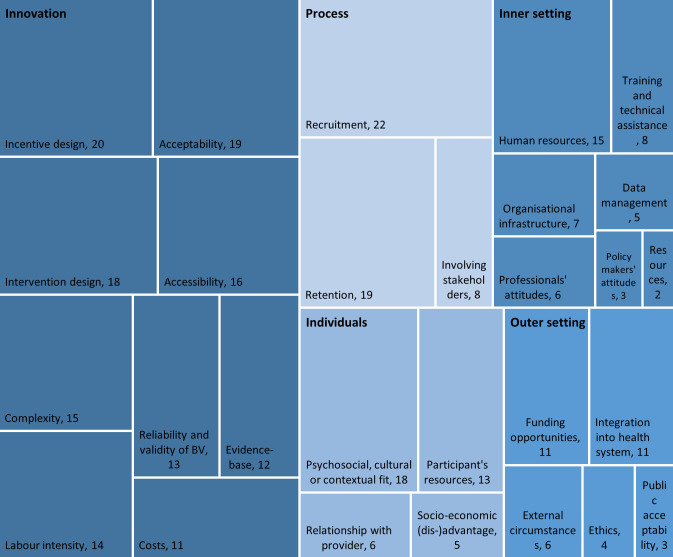
Overview of barriers and facilitators to the implementation of interventions with financial incentives. Size of the box corresponds to the number of studies in which the construct is mentioned as a barrier and/or facilitator. BV, biochemical validation.

The key factors influencing the implementation of financial incentives, as reported in the 37 included records, are shown in figure 2 and online supplemental figure 1. Online supplemental file 4 elaborates on these, providing supporting excerpts from the original reports. Barriers and facilitators are listed with references to the original research records, despite some barriers and facilitators being reported in secondary records.

#### Individuals domain

When individuals experienced a good fit of programmes with their cultural and personal circumstances, engagement with the intervention was facilitated, according to 14 out of 34 records. On the contrary, psychosocial and cultural barriers, such as feelings of bribery or distrust, negatively affected (expectant) parents’ willingness to engage. In 12 studies, a lack of resources impeded individuals from engaging with the intervention. The availability of time, money and transportation was essential, as interventions with incentives often require regular remote or face-to-face contact. Five studies reported that providing resources^50 79 81^ or using available resources^77 86^ for this purpose enabled engagement.

Staff state that they try to make the testing opportunity as accessible as possible, understanding that women may have transportation issues, variable work schedules, sick children, etc*.*^76^

#### Innovation domain

The design of the incentive system, as the core component of the interventions studied, largely determined the success of implementation. Authors suggested that since incentives for engagement were easier to implement than incentives contingent on validated abstinence, they could replace some of the incentives for abstinence in schemes.^49^ Incentivising engagement also increased attendance, including among participants who struggled to achieve and sustain abstinence.^81 82 87^ Delivery of incentives using a reloadable credit card^19 80^ or phone application^77^ offered opportunities for monitoring and limiting purchase choice. Moreover, incentives in the form of vouchers were most frequently considered acceptable (compared with products or cash) because the freedom of choice enhanced the rewards’ value and acceptability to recipients, while preventing harmful purchases^18 72 74^ and welfare payments from being cut.^19 72^ Regarding the overall intervention design, frequent, long-term support^75 85 88^ and involvement of close contacts^56 76 88^ was valued and suggested to enhance retention. Six studies highlighted the high costs of incentive programmes, while two rejected this barrier by putting costs in perspective relative to costly support alternatives and prevented expenses, demonstrating high cost-effectiveness.^74 89^ To limit costs, lower-cost schemes were recommended as well.^19 49 52 73 74 83^

Beneficiaries could only access fresh food and groceries through the program, and not alcohol or cigarettes. The use of the National Entitlement Card to redeem at the ASDA stores also avoided fraud and other pitfalls of handing out money or vouchers*.*^50^

Biochemical validation using exhaled breath CO had limitations in terms of reliability, as it only detects recent smoking, and in validity, as environmental factors can influence results. However, it was described as feasible more often than any other test method due to its simplicity, non-invasiveness and acceptability among participants,^18 19 51 73 78 90^ provided that testing frequency remains manageable.^77 78 86 90^ The possibility of gaming was reported to deter stakeholders from supporting implementation.^51 54^ Importantly, however, only around 4% of participants were found to falsely report their smoking status at inclusion according to estimates by Ierfino and colleagues.^53^ Using saliva, hair or blood for biochemical validation has been perceived as invasive,^19 56 78 80^ while frequent breath tests,^77 86 90^ compulsive watch-wearing^80^ or using multiple platforms^75^ were demanding. Receiving cotinine testing supplies by mail^80^ and exhaled breath CO testing^19 55^ were deemed acceptable. Cotinine^80^ and, especially, immediate exhaled breath CO^19 51 76 85 88 91 92^ test feedback were considered helpful to smoking cessation efforts. Overall, biochemical validation significantly contributed to the high demand for time and human resources, as mentioned in 11 studies.^18 20 48 53 54 56 73 79 83 88 89^ Testing at accessible locations, using technology for non-face-to-face delivery, and reducing its frequency were cited as facilitating implementation.

Although CO breath-testing has limitations as a tool for validation of smoking status, especially in terms of the persistence of CO on the breath, the method was simple to administer and had face validity for participants*.*^19^

#### Inner setting domain

Scarcity of available staff within organisations was reported as a barrier in 11 studies.^19 20 48 52 56 73 85 88 92–94^ Eight studies identified the need for training in the procedures of incentive programmes.^56 76 77 82 85 90 92 94^ Six studies noted barriers or facilitators related to organisational infrastructure,^48 52 76 81 85 92^ indicating that incorporation of incentive programme tasks (eg, screening) in standard procedures would facilitate implementation. Healthcare professionals’ attitudes had varying impact on implementation, with some studies identifying reluctance^53 56 82^ and others recognising the good reputation of the incentives.^48 54 82 88^ Policymakers’ reluctance to support incentive programmes was noted in three studies.^53 57 61^ Data management involved with testing and rewarding participants was frequently identified as a challenge.^20 50 76 77 95^

Challenges to feasibility were […] the extensive time involved with providing cessation support, including contacting women and travel to participants’ homes to verify smoking status*.*^62^

#### Outer setting domain

Securing long-term financial support for incentive programmes presents significant challenges.^19 51 72 74 76 92^ Successful strategies included: integrating incentives into existing interventions with stable funding or mobilising resources from local businesses, governmental agencies (eg, NHS, Medicaid or state funding), grants and health insurance companies.^19 48 52 73 74 81 92^ Apart from acquiring funds, integration into existing care models and national guidelines strongly facilitated implementation, but the process required perseverance.^92^ The implementation of incentive programmes could also be hindered by external factors, such as the COVID-19 pandemic.^51 85 91 93 94^ Lastly, addressing public scepticism^53 74 82^ and ethical concerns^56 68 72 80^ was considered essential.

The real hitch is: who should pick up the tab? Financial incentives make sense in closed health-care systems in which a single entity—such as the NHS—covers all of an individual’s health costs…^74^

#### Implementation process domain

Studies reported that engaging intervention recipients is a crucial process in implementation. Recruitment is supported by proactive and diverse contact methods,^53 92^ tailored to the context of both recruiter (eg, with time limitations) and target group.^56 73 92^ Retention barriers include difficulties in contacting participants, especially for biochemical validation, while frequent engagement^48 82 95^ and understanding participants’ context^71^ facilitate better retention.

The RA [Research Assistant] relied on a variety of approaches to contact women including face-to-face visits, E-mail, Facebook messaging, text messaging, and phone calls. Successful methods for contacting women were text messaging followed by phoning*.*^62^

Give It Up For Baby’ uses active recruitment of quitters via a care pathway, in which health professionals signpost women to their local community pharmacist for support*.*^50^

#### Implementation in populations with lower socioeconomic status

Subgroup analysis revealed differences in the distribution of barriers and facilitators to implementation between studies specifically focusing on populations with lower SES (n=18) and those that did not (n=19). Fewer studies involving lower SES participants reported on intervention design factors (6 vs 13 studies) and accessibility factors (6 vs 10 studies) facilitating implementation. No studies involving lower SES participants reported on the negative impact of COVID-19 (0 vs 6), and more such studies emphasised the positive impact of healthcare system integration (8 vs 3). These findings suggest variations in the factors influencing implementation success across different socioeconomic groups.

## Discussion

### Statement of principal findings

Financial incentives are a highly cost-effective approach to supporting smoking cessation among (expectant) parents.^12–14^ This is the first systematic review to comprehensively synthesise barriers and facilitators to their implementation. Major barriers include misalignment with participants’ context and resources, recruitment and retention challenges, high human resource demands and limitations in the reliability of biochemical validation. Key facilitators are ensuring acceptability, accessibility and feasibility of interventions with incentives, as well as securing funding and integration in existing services.

### Strengths and weaknesses of the study

This review provides an extensive analysis of the barriers and facilitators for the implementation of financial incentives, which is crucial for advancing their implementation across various settings.^96^ Ample evidence supports their effectiveness and cost-effectiveness, particularly during pregnancy, yet they remain underused in large-scale practice.^13 22 97^ A key strength of our review is the rigorous methodology we followed, using the TIDieR, Adams and CFIR frameworks,^37 44 59^ with a preregistered protocol on PROSPERO to enhance transparency. The inclusion of grey literature broadened the scope of evidence. We included a subgroup analysis for populations with lower SES, offering insight into differential factors affecting implementation. Limitations of the reviewed literature included a lack of reflection on implementation in general and a narrow geographical scope, potentially affecting generalisability to other settings. In the included records, incentives are embedded in varying interventions with varying evidence base for effectiveness.^67^ While this review gives an overview of the prevalence of each barrier and facilitator across studies, it leaves some uncertainty about their relative importance for successful implementation in specific contexts.

### Strengths and weaknesses in relation to other studies

The barriers and facilitators identified partly align with those reported in other reviews on smoking cessation programmes, while uniquely addressing issues specific to financial incentives and involving (expectant) parents. Consistent with a Cochrane review on smoking cessation programmes and studies on such programmes extended with incentives, we identified factors related to professionals’ attitudes, (human) resource availability, training, integration in routine care and administrative barriers.^9 12 98^ Our focus on implementation among (expectant) parents adds depth and distinct factors, including costs, funding, acceptability across various stakeholders and balancing feasibility and acceptability with efficacy regarding biochemical validation.

### Implications

Our findings provide valuable guidance for tobacco control policymakers, researchers, healthcare insurers and cessation support providers. Key implications are as follows. (A) Programmes should be tailored, flexible and designed in collaboration with the target population to address personal barriers and resource limitations. Recent literature and our subgroup analysis support the need for such tailored approaches, particularly for socioeconomically disadvantaged (expectant) parents.^9 14 50 68 97 99^ (B) Accessibility is crucial and may be enhanced through adopting digital technology^9 100 101^ or leveraging the regular standard healthcare visits of (expectant) parents.^102^ (C) The high demand for human resources can be mitigated through collaboration between healthcare professionals and smoking cessation counsellors.^9 103^ (D) Biochemical validation’s acceptability, costs, reliability, feasibility and acceptability should be balanced, as also outlined in a previous ethical framework.^23^ The use of biochemical validation and the low likelihood of deception^12 24^ can alleviate public concerns about unfairness. (E) Introducing programme components in a logical order and integration with existing interventions enhances implementation. Consistent with previous studies, we found that sustained and frequent support, also involving household members, improves engagement and acceptance.^104 105^ Our findings imply that using vouchers, including for engagement, social supporters and for participants who relapse, is most acceptable, aligning with discrete choice experiment outcomes.^106^ (F) Support for public funding and policy development hinges on the evidence base and, next, on public acceptability, which varies across cultures.^12 25^ Facilitators are early engagement of stakeholders,^96^ addressing their concerns about unintended consequences using evidence,^107^ non-paternalistic messaging^108 109^ and clear communication of cost-effectiveness,^110^ as well as emphasising that the costs are low compared with other interventions and to avoided healthcare costs.^110^ If programme costs still pose a barrier, alternative strategies may include using participant deposits (though this is expected to reduce uptake^12^), seeking voluntary donations (feasibility varies per country^28 51 52^) or using lower-value rewards, although the latter negatively affects cessation efforts.^83^

### Unanswered questions and future research

Future research should explore variation in these barriers and facilitators across diverse settings and populations and identify their relative impact on implementation outcomes such as adoption, implementation and sustainment.^111^ Additionally, prospective studies are needed to investigate how the findings from this study can support the implementation of interventions. Data on the implementation of incentive-based smoking cessation programmes for (expectant) parents is limited, with many studies in this review lacking comprehensive reporting and reflection on implementation strategies, and 21 out of 27 original research articles relying solely on quantitative methods. This limited insight hinders the translation of evidence into practice and raises questions about the role of fidelity in reported effectiveness, as pointed out in a Cochrane review demonstrating the efficacy of financial incentives.^9 112^ To improve understanding, future studies should prioritise process evaluations, mixed-methods and qualitative methods. Interviewing authors and intervention providers could also provide valuable perspectives.

As a remaining unanswered question, we lacked data allowing us to analyse implementation during the preconception and postnatal phases. Expanding the target population beyond pregnant women could positively affect more children, reduce stigma and negative perceptions associated with targeting only pregnant women and provide a longer time frame for intervention beyond the 9 months of pregnancy.

## Conclusions

This systematic review offers new insights into the challenges and opportunities of implementing financial incentives for smoking cessation among (expectant) parents. As their cost-effectiveness is well-documented,^12–14^ attention must now shift to optimising implementation. Key considerations include ensuring appropriateness, accessibility, resource allocation and integration with existing policies. Given the ongoing and substantial harm caused by smoking, especially around children, timely implementation of financial incentive interventions is crucial.

## Supplementary material

10.1136/tc-2024-059198 online supplemental file 1

## Data Availability

All data relevant to the study are included in the article or uploaded as supplementary information.
