# Supplementary material for: Financial incentives for smoking cessation among (expectant) parents: a systematic review of facilitators and barriers to implementation
Source: Tob Control. 2025 Apr 22;35(4):e059198. doi: 10.1136/tc-2024-059198 (PMC13479641; doi:10.1136/tc-2024-059198)
Supplement: Supplementary file 1 [file tc-35-4-s001.docx]

Supplementary files

## Supplementary file 1. Protocol

**Facilitators and barriers to the implementation of incentives within smoking cessation programmes for (expectant) parents: a mixed-methods systematic review protocol**

*Linda van der Spek, Leonieke J. Breunis, Linda Bauld, Hans van Kippersluis, Erwin Ista, Tessa Scheffers-van Schayck, Jasper V. Been*

**Citation**

Linda van der Spek, Leonieke J. Breunis, Linda Bauld, Hans van Kippersluis, Erwin Ista, Tessa Scheffers-van Schayck, Jasper V. Been. Facilitators and barriers to the implementation of incentives within smoking cessation programmes for (expectant) parents: a mixed-methods systematic review protocol. PROSPERO 2023 CRD42023407648 Available from: <https://www.crd.york.ac.uk/prospero/display_record.php?ID=CRD42023407648>

**Review question**

What are the barriers and facilitators to the successful implementation of financial incentives as part of smoking cessation programmes for (expectant) parents?

**Searches**

- Search date: 15-08-2022.
- Bibliographic databases: EMBASE (1971 - Present), MEDLINE (OVID) (1946 - Present), the Cochrane Central Register of Controlled Trials (CENTRAL, 1992 - Present), Web of Science (1975 - Present), Google Scholar, PsycINFO (1806 - Present), CINAHL (1982 - Present), and EconLit (1886 - present).
- Reference lists of reviews related to the topic and of included studies and their citations will be screened using Scopus, following Bramer (Bramer, 2018).
- Grey literature (e.g. opinion papers, books, guidelines, policies, dissertations, as defined by Luxembourg definition) will be systematically searched using the Google search engine.
- No restriction will be imposed on language or publication period. Studies published in other language than English will be translated using Google Translate and, if necessary, a translator will be consulted.
- The searches will be re-run just before the final analyses and any further studies identified will be retrieved for inclusion.

Additional search strategy information can be found in the attached PDF document (link provided below).

**Types of study to be included**

We will include any study design. Regarding publication type, we will include full journal articles of primary research, editorials, commentaries and book sections, as well as grey literature. We will exclude conference abstracts, letters, news releases, reviews and study protocols.

**Condition or domain being studied**

Smoking among (expectant) parents.

**Participants/population**

People with a wish to conceive, pregnant women and their partners, and parents of children aged 0-18 years.

**Intervention(s), exposure(s)**

Implementation of financial incentives as part of a smoking cessation support programme to reward individuals for cessation and abstinence.

Implementation is defined as the constellation of processes aimed at getting an intervention into use within an organization or setting (Damschroder et al., 2009; Pfadenhauer et al., 2017). Financial incentives are defined by Adams et al. (2012) as cash and cash-like rewards, including (vouchers for) goods and services, contingent on smoking cessation or abstinence.

**Comparator(s)/control**

Not applicable.

**Context**

Research in a high income country as defined by the World Bank.

**Main outcome(s)**

The main outcome is the description and reflection on (barriers and facilitators, or synonyms thereof, to) actual or proposed implementation.

**Additional outcome(s)**

None.

**Data extraction (selection and coding)**

Data management:

- Retrieved articles will be exported to EndNote for screening purposes, and subsequently to AtlasTi for data extraction and analysis.

Selection process:

- Two researchers (LJB and LS) will independently screen titles and abstracts. All articles deemed eligible based on inclusion and exclusion criteria will be obtained and screened independently in full by two researchers. Disagreement occurring during screening of titles and abstracts or full-texts will be resolved with discussion, if necessary involving a third member of the review team.

Data extraction process:

- A customised data extraction form will be used for this review, covering study characteristics, items from the TIDieR checklist (describing interventions), items from the framework for documenting on financial incentives (Adams et al., 2014), and the reported implementation barriers and facilitators, effectiveness, costs and sustainability.
- Microsoft Excel will be used to store extracted data. AtlasTi will be used for coding.
- Two researchers will extract data independently (LJB and LS). Inconsistencies between extracted items will be discussed to reach consensus, or otherwise a third researcher will be consulted (JB).
- When the researchers are confronted with substantial unclarities or incompleteness regarding extracted data, data extraction files will be shared with study authors or intervention owners via e-mail, for feedback, additional information and verification.

**Risk of bias (quality) assessment**

- The Mixed Methods Appraisal Tool (MMAT-version 2018) will be used to assess quality of each study. The MMAT is relevant to the methodologies of five study types, namely qualitative studies, quantitative randomised controlled trials, quantitative non-randomised studies, quantitative descriptive studies, and mixed-methods studies. It is considered a particularly good fit for process-oriented intervention research in public health context (Hong et al., 2018). A methodological rating of “yes,” “no,” or `”can’t tell” will be assigned to two general aspects and to five aspects specific to the study type, to produce an overall score (Hong et al., 2018).
- The main interest of our study lies in self-reported barriers and facilitators to implementation, preferably in a real-world setting. Therefore, high methodological quality as measured by the MMAT is not of direct relevance to the interpretation of the main outcomes, which is also why grey literature will be included. In order to retrieve a diverse and substantial literature base, the quality assessment will not be used to exclude any studies, but will instead only inform the interpretation of outcomes.

**Strategy for data synthesis**

- Study characteristics will be reported in tabular and narrative form.
- First, narrative deductive thematic synthesis of qualitative data will be conducted. Second, quantitative data will be mapped to the qualitative review findings.
- Thematic synthesis will be based on the ‘Consolidated Framework for Implementation Research’ (CFIR) key constructs: intervention characteristics, outer setting, inner setting, characteristics of the individuals, and the implementation process.

**Analysis of subgroups or subsets**

Since smoking is a major cause of health disparities due to the substantially higher smoking prevalence among socioeconomically disadvantaged people, including (expectant) parents, additional understanding of implementation strategies tailored to the needs of socioeconomically disadvantaged (expectant) parents will be provided through sub-group analysis and synthesis (European Commission, 2014; Krieger et al., 1997; Mackenbach, 2014; Scheffers-van Schayck et al., 2022).

Supplementary file 2. Search strategy

Scientific literature

**medline**

(exp "Tobacco Use Cessation"/ OR exp Smoking Cessation Agents / OR exp "Tobacco Use"/ OR exp "Tobacco Use Disorder"/ OR Nicotine / OR (smoking OR smoke* OR tobacco OR cigarette* OR nicotin*).ab,ti.) AND (exp Pregnancy / OR Pregnant Women / OR exp Prenatal Exposure Delayed Effects / OR (pregnan* OR prepregnan* OR preconception* OR pre-conception* OR prenatal* OR pre-natal* OR parent* OR father* OR mother* OR Maternal* OR paternal* OR child-wish OR childwish OR gestation* OR antenatal* OR ante-natal*).ab,ti.) AND (Reimbursement, Incentive / OR Token Economy / OR (incentiv* OR reward* OR voucher* OR (token ADJ3 (econom* OR reinforce*))).ab,ti.) NOT (exp animals/ NOT humans/)

**Embase**

('smoking cessation'/de OR 'smoking cessation program'/de OR 'smoking cessation agent'/de OR 'tobacco use'/exp OR 'tobacco dependence'/de OR nicotine/de OR (smoking OR smoke* OR tobacco OR cigarette* OR nicotin*):ab,ti) AND (pregnancy/exp OR 'prepregnancy care'/de OR 'pregnant woman'/de OR 'parental behavior'/exp OR 'parental smoking'/exp OR parent/exp OR 'prenatal exposure'/exp OR (pregnan* OR prepregnan* OR preconception* OR pre-conception* OR prenatal* OR pre-natal* OR parent* OR father* OR mother* OR Maternal* OR paternal* OR child-wish OR childwish OR gestation* OR antenatal* OR ante-natal*):Ab,ti) AND (incentive/de OR 'social incentive'/de OR 'economic incentive'/de OR 'monetary reward'/de OR 'token economy'/de OR 'voucher program'/de OR (incentiv* OR reward* OR voucher* OR (token NEAR/3 (econom* OR reinforce*))):Ab,ti) NOT ([animals]/lim NOT [humans]/lim) NOT [conference abstract]/lim

**Web of science**

TS=(((smoking OR smoke* OR tobacco OR cigarette* OR nicotin*)) AND ((pregnan* OR prepregnan* OR preconception* OR pre-conception* OR prenatal* OR pre-natal* OR parent* OR father* OR mother* OR Maternal* OR paternal* OR child-wish OR childwish OR gestation* OR antenatal* OR ante-natal*)) AND ((incentiv* OR reward* OR voucher* OR (token NEAR/2 (econom* OR reinforce*))))) NOT DT=(Meeting Abstract OR Meeting Summary)

**Cochrane**

((smoking OR smoke* OR tobacco OR cigarette* OR nicotin*):ab,ti) AND ((pregnan* OR prepregnan* OR preconception* OR pre NEXT conception* OR prenatal* OR pre NEXT natal* OR parent* OR father* OR mother* OR Maternal* OR paternal* OR child NEXT wish OR childwish OR gestation* OR antenatal* OR ante NEXT natal*):Ab,ti) AND ((incentiv* OR reward* OR voucher* OR (token NEAR/3 (econom* OR reinforce*))):Ab,ti)

**CINAHL**

(MH " Smoking Cessation Programs +" OR MH Smoking Cessation + OR MH Tobacco Use Cessation Products + OR MH Smoking+ OR MH Nicotine OR TI(smoking OR smoke* OR tobacco OR cigarette* OR nicotin*) OR AB(smoking OR smoke* OR tobacco OR cigarette* OR nicotin*)) AND (MH Pregnancy + OR MH Expectant Mothers OR MH Prenatal Exposure Delayed Effects + OR TI(pregnan* OR prepregnan* OR preconception* OR pre-conception* OR prenatal* OR pre-natal* OR parent* OR father* OR mother* OR Maternal* OR paternal* OR child-wish OR childwish OR gestation* OR antenatal* OR ante-natal*) OR AB(pregnan* OR prepregnan* OR preconception* OR pre-conception* OR prenatal* OR pre-natal* OR parent* OR father* OR mother* OR Maternal* OR paternal* OR child-wish OR childwish OR gestation* OR antenatal* OR ante-natal*)) AND (MH Reimbursement, Incentive OR TI(incentiv* OR reward* OR voucher* OR (token N2 (econom* OR reinforce*))) OR AB(incentiv* OR reward* OR voucher* OR (token N2 (econom* OR reinforce*)))) NOT (MH animals+ NOT MH humans+)

**PsycINFO**

(exp Smoking Cessation / OR exp Tobacco Smoking / OR Nicotine / OR (smoking OR smoke* OR tobacco OR cigarette* OR nicotin*).ab,ti.) AND (exp Pregnancy / OR exp Prenatal Exposure / OR (pregnan* OR prepregnan* OR preconception* OR pre-conception* OR prenatal* OR pre-natal* OR parent* OR father* OR mother* OR Maternal* OR paternal* OR child-wish OR childwish OR gestation* OR antenatal* OR ante-natal*).ab,ti.) AND (	Incentives / OR Monetary Incentives / OR Token Economy Programs / OR (incentiv* OR reward* OR voucher* OR (token ADJ3 (econom* OR reinforce*))).ab,ti.) NOT (exp animals/ NOT humans/)

**EconLit**

(TI,AB(smoking OR smoke* OR tobacco OR cigarette* OR nicotin*)) AND (TI,AB(pregnan* OR prepregnan* OR preconception* OR pre-conception* OR prenatal* OR pre-natal* OR parent* OR father* OR mother* OR Maternal* OR paternal* OR child-wish OR childwish OR gestation* OR antenatal* OR ante-natal*)) AND (TI,AB(incentiv* OR reward* OR voucher* OR (token N/2 (econom* OR reinforce*))))

**Google scholar**

smoking|smoke|tobacco|cigarette|nicotine pregnancy|pregnant|prepregnancy|preconception|prenatal|childwish|gestational|antenatal incentive|incentives|reward|voucher|"token economy"|reinforcement

Grey literature

**Google**

intitle:smoking|intitle:smoke|intitle:tobacco|intitle:cigarette|intitle:nicotine pregnancy|pregnant|prepregnancy|preconception|prenatal|childwish|gestational|antenatal incentive|incentives|reward|voucher|"token economy"|reinforcement filetype:pdf -doi -pmid

allintitle:smoking|smoke|tobacco|cigarette|nicotine pregnancy|pregnant|prepregnancy|preconception|prenatal|childwish|gestational|antenatal incentive|incentives|reward|voucher|"token economy"|reinforcement -doi -pmid

Supplementary file 3. Risk of bias (MMAT)

| Mixed-methods studies | Clear research questions | Appropriate data | Adequate rationale | Effective integration | Adequate interpretation | Divergences addressed | Adhered to quality criteria | Score* | Comments |
| --- | --- | --- | --- | --- | --- | --- | --- | --- | --- |
| Askew et al, 2019 | Yes | Yes | Yes | Yes | Yes | Yes | Yes | 100% | Each criterium is met, no comments. |
| Breunis et al, 2023 | Yes | Yes | Yes | Yes | Yes | Yes | Yes | 100% | Each criterium is met, no comments. |
| Kroder et al, 2022 | Yes | Yes | Yes | Yes | Yes | Can't tell | No | 60% | No adequate addressing of divergences between quantitative and qualitative results, since the discussion section does not include a clear integration of both quantitative and qualitative findings. Regarding adherence to quality criteria of both quant and qual methodologies: The study seems to adhere to the quality criteria for quantitative research, specifically for feasibility studies and RCTs, but crucial information on the qualitative part lacks. |
| Radley et al, 2013 | Yes | Yes | Yes | No | Yes | Can't tell | Yes | 60% | Effective integration: The process evaluation helped to explain variations in retention and quit rates achieved by the scheme, but, on the other hand, the indicators from the qualitative studies that explain why certain parts of the intervention did or did not work are not discussed and many of the quantitative study's conclusions were not clearly tested in the qualitative study. |
| Allison et al, 2023 | Yes | Yes | Yes | Yes | No | No | No | 40% | Evaluates effectiveness through quantitative metrics (operational metrics) and qualitative insights (semi-structured interviews), which is presented with adequate rationales. Integration of quant and qual insights is sufficient, insights are offered into how they explain each other ("It appears that the qualitative data contradicts the quantitative data") but could have gone deeper. Interpretation is still lacking: while there is an intention to interpret the outputs, this process is reported to be still in progress. Therefore, divergences could also not be addressed adequately (yet). Also, quality criteria are not fully adhered to due to the incompleteness of analyses and the lack of a methods section. |
| Quantitative nonrandomised studies | Clear research questions | Appropriate data | Representative population | Appropriate measurements | Complete outcome data | Accounted for confounding | Exposure as intended | Score | Comments |
| Gadomski et al, 2011 | Yes | Yes | Yes | Yes | Yes | Yes | No | 80% | Representative population, however, the programme did not serve people with heavier smoking patterns well. Complete outcome data with 23% lost to follow-up (159/708). No full exposure as intended, since prenatally, 58% (410/708) of women received all four sessions of the intervention. |
| Ierfino et al, 2015 | Yes | Yes | Yes | Yes | Can't tell | Yes | Yes | 80% | Can't tell whether outcome data was complete, as drop-out was not reported, but the proportion of those enrolled who made a quit attempt was 60%. Exposure as intended, though target of making three attempts to contact people who smoke not achieved. |
| Kurti et al, 2020 | Yes | Yes | No | Yes | Can't tell | No | Yes | 40% | The effort required to enrol and exclusion of women who reported frequent marijuana use and being unwilling to quit may have reduced representativeness of the population, though a relatively low proportion of women was excluded for the latter reasons (~5%). |
| Miller et al, 2017 | Yes | Yes | Can't tell | Yes | Yes | No | No | 40% | Not accounting for confounding, though authors acknowledge that 'Our population had high rates of buprenorphine/methadone use and opiate drug abuse as demonstrated by urine drug testing at delivery in both the control and CM groups. This altered the birth outcome data." No exposure as intended: Only 2/11 participants submitted all of the requested CO readings, and 5/11 patients with over 80% compliance with the program. "Adherence to the CM programme was lower in our programme (55%) when compared to other programmes which may be a reflection of our high-risk population." |
| Passey and Stirling, 2018 | Yes | Yes | Yes | Yes | Yes | No | Yes | 80% | The population was considered representative, as "There were no significant differences in age, Aboriginal status, gestation or smoking status between women who enrolled and those who declined." Accounting for confounding not reported. Regarding exposure as intended, not everybody joined the group sessions, but those were optional. |
| Too et al, 2021 | Yes | Yes | Yes | Yes | Yes | No | Yes | 80% | Complete outcome data, as 133 of 739 and 128 of 672 were missing, so over 60% of outcome data available. Confounders not accounted for: "Random allocation was not used so that presumptions about even distribution of unmeasured confounding variables between groups cannot be made." |
| Wen et al, 2019 | Yes | Yes | Can't tell | Yes | Yes | No | Yes | 60% | Can't tell whether population was representative, authors state that selection bias may have occurred, that the sample size is relatively small, and that generalizability might be limited because participants were only recruited from a small geographical area. However, the mean age was close to the national average. 70% retention so outcome data is considered complete. Authors do not account for confounding. |
| Zhang et al, 2017 | Yes | Yes | Yes | Yes | Yes | Yes | Yes | 100% | Representative population, though participants were younger, non-Hispanic black, less often married, and more often lower SES than non-participants. Appropriate measurements, though different from most studies in our review: they measured the effect of going to sessions on birth outcomes, instead of directly the effect of incentives on birth outcomes/smoking cessation. Complete outcome data: only 0.6% missing data (69/12434). |
| Quantitative descriptive studies | Clear research questions | Appropriate data | Relevant sampling | Representative sample | Appropriate measurements | Nonresponse bias low | Appropriate analysis | Score | Comments |
| Jackson et al, 2023 | Yes | Yes | Yes | Yes | Yes | Yes | Yes | 100% | Risk of nonresponse bias and representativeness of the population: Half the potentially interested clients were enrolled, and almost half of those who did enrol were retained until the 12-week postpartum follow-up, but comparisons with studies in similar populations are difficult due to heterogeneity in contexts, populations and approaches. Recruitment may also have been hindered by the perceived burden of self-recording and using multiple digital platforms. At the same time, uptake and acceptability were deemed acceptable. |
| Valencia et al, 2020 | Yes | Yes | No | Yes | Yes | No | N/A | 40% | Small sample, so no relevant sampling. Measurements are appropriate but would have been even more appropriate if an additional qualitative study was conducted. Appropriate analysis is not applicable, since no statistical analyses were applied due to limited sample size. |
| Yon et al, 2022 | Yes | Yes | Yes | Can't tell | Yes | No | No | 40% | No population descriptives available to show whether the sample is representative of the target population. "Out of approximately 256 births to Rutland County women who smoked, we enrolled 20 women (13 from WIC; seven from RWH)." Not possible to determine whether these 20 out of 256 are representative. Risk of nonresponse bias is not considered low, since discussing smoking is not standard practice for staff and depends on other priority topics. Analyses (and the lack thereof) are not clearly justified, so this is not considered appropriate analysis. |
| Randomised Controlled Trials | Clear research questions | Appropriate data | Appropriate randomisation | Baseline similarity | Complete outcome data | Blinding | Exposure as intended | Score | Comments |
| Alaniz et al, 2019 | Yes | Yes | Yes | Yes | No | No | No | 40% | Appropriate randomisation: Group assignment was random, blocking by region and race (3 geographic regions with each a list of random allocations (intervention vs control) for white and for non-white women). No statistically significant differences between control and intervention participants on any of the 31 baseline questions, with one exception: quit attempt in the past year (83.5% (I) vs 71.7% (C); P=0.04), but the MMAT manual only considers baseline similarity to be lacking if imbalance in key prognostic factors is unlikely to be due to chance. No complete outcome data, since 98 women (53.0%) completed the 6-month postpartum visit. It was not possible to conceal group assignment from field staff: data collection at the same time as intervention provision. No exposure as intended, since the level of engagement was rated as low for 23.9% of these women, medium for 30.4%, and high for 45.7%. Difficulty to reach and complete appointments rated as easy for 43.5% of intervention participants, medium for 26.1%, and high for 26.1%. |
| Baker et al, 2018 | Yes | Yes | Yes | Yes | Yes | No | Can't tell | 60% | Appropriate randomisation since separate computer determined randomisation tables were created based on race (White/non-White) and county with proportional randomisation (1:1) into the incentive and control conditions. Complete outcome data for the primary outcome, as a total of 316 of 509 (37.9%) control condition participants had missing data and a total of 145 of 505 (28.7%) incentive condition participants had missing data. No blinding and cannot tell whether exposure was as intended, only a high no-show rate was reported for prebirth clinic visits. |
| Donatelle et al, 2000 | Yes | Yes | Can't tell | Yes | Yes | Can't tell | Yes | 60% | Randomisation and blinding procedures were not described. |
| Glover et al, 2015 | Yes | Yes | Yes | Can't tell | No | No | Can't tell | 20% | Randomisation was appropriate: "Immediately after baseline data had been recorded, participants were randomised in a 1:1 ratio to one of three arms using envelope randomisation prepared by a statistician." Can't tell whether there was baseline similarity, as participant demographics are given, but differences were not statistically tested. No complete outcome data: of the 24 women who agreed to participate, 15 withdrew and 12 were lost within 8 weeks, giving a retention rate of 37.5%. |
| Gulliver et al, 2004 | No | Can't tell | Can't tell | Can't tell | Can't tell | Can't tell | Can't tell | 0% | The article lacks the crucial reporting on study procedures, even the screening questions are answered no and can't tell. |
| Harris and Reynolds, 2015 | Yes | Yes | Yes | Yes | Yes | Can't tell | Yes | 80% | Randomised appropriately to either the CM or SCHB treatment group using a random number generator available via the Internet (Research Randomiser, n.d.). "Participants across conditions were similar on all variables, including length of pregnancy and readiness to quit smoking at enrolment (p > .05)." Complete outcome data since 68.2% of samples was provided. The paper lacks reporting on blinding procedures. Participants provided an average of 68.2% of their required, twice-daily breath samples, which was considered exposure as intended. |
| Heil, 2008 | Yes | Yes | Can't tell | Yes | Yes | No | Yes | 60% | Can't tell whether randomisation was performed appropriately: "Randomisation was stratified based on the clinic where participants received their prenatal care." "No significant differences in socio-demographics or smoking characteristics … at the study-intake assessment" Complete outcome data and exposure as intended, since "Compliance with periodic assessments was relatively high (83–95%)". Outcome assessors also provided incentives, so no blinding. |
| Higgins et al, 2014 | Yes | Yes | Can't tell | Yes | Can't tell | Can't tell | Can't tell | 20% | Only reporting "Randomly assigned", so can't tell whether procedures were appropriate. Baseline similarity (RCV participants worked outside the home more often and reported higher mean ratings of stress compared to the CV but not the NCV conditions. However, this is not significantly correlated with smoking abstinence.) Authors do not report drop-out rates. Can't tell whether there was appropriate blinding, as there was on-site biochemical validation of smoking status followed by provision of incentives in the treatment group, but, on the contrary, evaluation of birth outcomes was done by blinded evaluators. |
| Joyce et al, 2021 | Yes | Yes | Can't tell | Can't tell | Yes | Can't tell | Yes | 40% | Complete outcome data (67% retention). Exposure considered 'as intended' since authors report that feasibility and acceptability were fair. |
| McMeekin et al, 2023 | Yes | Yes | Can't tell | Can't tell | Can't tell | Can't tell | Can't tell | 0% | Can't tell whether outcome data was complete. Regarding cost-effectiveness data: 610 complete cases, 330 participants were missing either total costs or late-pregnancy QALYs, so outcome data for 65% of the cases. But unknown for effectiveness data. This paper reports on a secondary (cost-effectiveness) analysis and lacks crucial information for quality analysis. However, such information may be reported in the primary paper. |
| Ondersma et al, 2012 | Yes | Yes | Yes | Yes | Yes | No | Yes | 80% | Computer randomisation is considered appropriate. "No significant differences between conditions on any of the baseline characteristics, although one variable (minority vs. nonminority race) was below p = .10 and so was controlled for in subsequent analyses." 85% retention so complete outcome data. Partial blinding, as research staff were aware of CM-Lite status. Exposure as intended, considering that participants were free to test or not. |

*The first two items are screening questions, which are not considered for calculation of the overall score.

Supplementary file 4. Facilitators and barriers

*Facilitators and barriers to implementation in the individual’s domain.*

| Theme | Barriers | Facilitators | Quote(s) |
| --- | --- | --- | --- |
| Psychosocial, cultural or contextual fit | Psychosocial (19,51,56,83,85), cultural (56,75,78,83), or contextual barriers (e.g., relocation) (19,48,81,86,88,92). | Personalised approach (19,48,51,81,87,90,92), cultural fit (48,54,56,68,74,75,83,90,94), or meeting social needs (19,48,76,90,94). | "…focused on meeting each individual’s needs, and accounted for the social determinants of health including historical antecedents, community norms and cultural strengths." (48) |
| Participant’s resources | Transportation (49,76–78,83,86,90,92,93), time (82,83,86,88,90,92,93), childcare (76,82,83,92), phone credit/service (48,78) or money (78). | Resources are provided (19,76,78) or already available (i.e., smartphone, electricity and internet access) (86,88). | “Lack of childcare and transportation were also identified as barrier to treatment attendance.” (76) |
| Socio-economic (dis-) advantage | Populations with a lower SES may be more difficult to recruit and retain (52,71), due to disparities in technology use (86) and in quality and use of care (19,52). | Higher receptiveness to incentives in populations with a lower SES (74). | "Thus, recruitment methods that leverage digital forms to reach pregnant women who smoke may inadvertently target a narrower, more affluent segment of this population than intended." (86) |
| Relationship with provider |  | Relationship between participant and provider (19,48,51,56,90) | "Rapport and engagement with women (…) were considered to be critical..." (90) |

*Facilitators and barriers to implementation in the innovation domain.*

| Theme | Barriers | Facilitators | Quote(s) |
| --- | --- | --- | --- |
| Incentive design | Content of incentive may be of limited value for specific groups (19). Delivering incentives to a group causes negative group pressure (51). The high value of incentives may be too costly for scale-up (81). | Feasible and acceptable: products (75), lottery tickets (77), or vouchers (18,19,94,56,72,77,82,83,85,90,93), incentives for engagement (49,76,80,81,85), lower value incentives (89), using a reloadable credit card (19,82), or phone application (86) for delivery, and incentives for social supporters and/or participants who relapsed (18,56,92,94). | “This level and schedule of reinforcement was chosen for its feasibility relative to programmes with higher incentive levels, (…) and for its relative simplicity.” (89) |
| Reliability and validity of biochemical validation | The detectable timeframe of breath CO (19,49,75,91) is limited, environmental factors affect test results (i.e., air pollution, second-hand smoke, and cannabis-use) (86,91,92). Potential (51,54) and actual gaming (though minor) (53) impacts the willingness to implement. | Cotinine (52,85), CO (91) or thiocyanate (52) testing is considered accurate. Anabasine (53) and smartwatch (82) results are not affected by Nicotine Replacement Therapy. A higher CO-cut off point minimises false positives (86). The absence of evidence of substantial gaming (53,75) supports implementation. | "Breath CO, which was used in this trial, has a relatively brief half-life; serum cotinine might have been more sensitive to detecting temporally remote smoking." (49) |
| Intervention design |  | Providing incentives as an adjunct to existing support facilitates implementation (56,68). Authors advocate for flexible, holistic, tailored, comprehensive, consistent and structured designs (19,48,90,92), similar to the efficacy trial design (53), additional (behavioural) support (53,70,81,90), and frequent and long-term support (81,90,93), which is facilitated by technology-based delivery (81,83,86,91,93). Implementation was facilitated by provision by teaming providers (90) or by dedicated specialists (71,74). Offering support to household members is valued (56,83,90), facilitates engagement (92,94,96) and is facilitated by willingness of partners (76,90) and remote programmes (73). | “Consistency is another important consideration to ensure different intervention components do not contradict with each other.” (92)  "Incentives seem most effective when provided as an adjunct to psychosocial counselling, and as such could be integrated into existing services to support cessation." (68) |
| Acceptability | Frequent biochemical validation is demanding (74,81,86,88) and watch-wearing or providing hair or blood samples are invasive (19,56,82,91). | Participants considered cotinine (82) and CO (19,55) testing acceptable and helpful (19,51,65,73,83,90,93). Low frequency (49,73,75,89) and non-face-to-face delivery (81) of BV reduces participant burden and increases acceptability. | “…it gave immediate feedback. (…) Use of other methods such as salivary or blood testing of cotinine were felt to have significant drawbacks because of their invasive nature." (19) |
| Accessibility | Frequent on-site validation reduces accessibility (86). | Offered at an accessible location (e.g., at home, or integrated into locations already visited) (56,74,94,75,76,78,79,83,86,87,93), at flexible locations and/or times (74,77,82,83,87,91), or using technology to increase accessibility (75,81,83,86,88,93). | ""Study results demonstrating the efficacy of smartphone-based financial incentives highlight the capacity for technology to expand the reach of this efficacious, evidence-based treatment..." (86) |
| Complexity | Complexity (53,83,86,90) or issues with technology (86,88,91) | Practicability of testing CO (18,19,51,74,75,91), cotinine (52,74,82), thiocyanate (52), or remotely (81,82,91), and of provider devices (56) or the overall programme (49,90). | "...the intervention is too complex and time-consuming to integrate into routine obstetrical care." (61) |
| Labor intensity | Interventions are time- and labour-demanding (18,20,90,48,53,54,56,75,78,79,89). | The limited intensity of interventions can facilitate implementation (73,81,89). | "Challenges to feasibility were (…) the extensive time involved with (..) travel to participants’ homes to verify smoking status." (62) |
| Costs | Costliness of the incentive schemes (18,54,78,79,81,89) or of salivary cotinine analysis (52). | Implementing lower-cost schemes (19,49,52,75,77,89) or comparing costs to other treatment options and prevented health expenses (77,79). Salivary thiocyanate (52) and breath CO (18,19,75) are inexpensive tests. | "...“it’s quite a cheap intervention”, he says — and cheaper still if it improves the long-term health of mothers and their babies." (77) |
| Evidence-base | Lack of evidence reduces approval (19,80,83) and generating evidence presents challenges (88). | Sufficient evidence of (cost-)effectiveness is available (19,53,68,81,86,89,94) to support implementation. | "Demonstrating the cost-effectiveness (…) may be the final arbiter of whether state Medicaid programmes will support incentives." (86) |

*Facilitators and barriers to implementation in the inner setting domain.*

| Theme | Barriers | Facilitators | Quote(s) |
| --- | --- | --- | --- |
| Human resources | Limited human resources available (19,20,95,48,52,56,75,87,90,93,94). | Dedicated staff (74) and overall staff availability (20,53,71,83,94). | "Challenges to feasibility were […] and the extensive time involved with providing cessation support, including contacting women and travel to participants’ homes to verify smoking status." (62) |
| Training and technical assistance | Training needs (83,86,93,95). | Provision of technical assistance (83,94) and (ongoing) training in delivering incentive schemes (56,74,83,85,93–95). | "Managing these contingencies, associated record keeping, and biochemical verification methods can be challenging for personnel without training to implement with fidelity." (86) |
| Attitudes of healthcare professionals | Reluctance (53,56,85). | Professionals being positive about incentive programmes (48,54,85,90), and using education to increase their acceptance (85). | "Some professionals were hesitant at first, as they thought the incentives were unfair to the non-smoking pregnant women." (85) |
| Organizational infrastructure | Standard workflows offering few opportunities to recruit (52,87,93) and not covering universal screening and referral (76). Conflicting priorities and imperfect relationships between staff and cessation counsellors (87). | Integration in standard procedures (83,85,87,94), with efficient operationalization (83) and colocation of services (87). | "Although practices wanted to refer participants, few had adequate procedures in place to do so.” (76) |
| Attitudes of policymakers | Reluctance (53) and lack of a leader in implementation (93). |  | "A greater challenge may be to address policy makers and public scepticism about such schemes, but monitoring and reporting on gaming can contribute to that." (57) |
| Data management | Data management is challenging (19,20,83,86,96). | Training, tracking tools, and quality assurance are beneficial (83). | "Staff need to be trained (…) [in] data management (specifically entering data into the database), and tracking incentives to ensure consistence." (83) |
| Resources | Lack of space for equipment (56) | Necessary resources are provided to sites (94). | "Interested sites participate in a training programme and receive toolkits, outreach training, CO monitors, vouchers and ongoing monthly technical assistance." (64) |

*Facilitators and barriers to implementation in the outer setting domain.*

|  | Barriers | Facilitators | Quote(s) |
| --- | --- | --- | --- |
| Funding opportunities | Securing long-term financial support from local entrepreneurs and stakeholders (51,83) or from public funds in the absence of a single-payer healthcare system (like the NHS) (19,51,72,77,83,94). | Integration into an existing intervention with stable funding (49) or mobilization of funds from local business (19,52,75,76), governmental agencies, grants, or health insurance companies (77,94). | "In countries such as the United States, which lack a single-payer health-care system, contingency management can be more difficult to implement." (77) |
| Integration into health system | Integration, as a multi-agency transition process, is a long-term process (94). | Embedding them within existing care models (19,48,49,53,68,75,87,94), national guidelines and routine care (51,53,72,83,85,94). | "The incentive intervention was made available as an adjuvant to an ongoing state-supported intervention programme (..). Thus, the incentive programme was compatible with real world prebirth and post birth care." (49) |
| External circumstances | The Covid-19 pandemic impeded implementation (51,73,81,87,93,95). |  | "...our offices closed and/or restricted in-person interactions because of the Covid-19 pandemic. Our brief counselling sessions transitioned outdoors with drive-through cotinine testing." (93) |
| Public acceptability | Scepticism (53,77,85) |  | "A greater challenge may be to address policy makers and public scepticism about such schemes…" (57) |
| Ethics | Particularly in vulnerable populations, there is risk of coercion or exploitation (56,72). Addressing smoking rather than the underlying inequality is dubious (56,82). | Ethical consideration benefits appropriate implementation (68). There is no specific evidence of adverse or unintended consequences of incentives (72). Ethical concerns are outweighed by the benefits and counterarguments (72). | "Appropriate ethical consideration is also essential." (68)  “There was considerable concern about (…) potentially exploiting economic desperation.” (56) |

*Facilitators and barriers to implementation in the process domain.*

|  | Barriers | Facilitators | Quote(s) |
| --- | --- | --- | --- |
| Recruitment | Lengthy enrolment process requiring biochemical validation and web access (81,86), indirect recruitment by healthcare professionals causing delay (56,75,79). Professionals being hesitant or forgetting to refer (85), lacking confidence (93), time or procedures to refer (48,76), referring in a not so welcoming way (75), narrowly focusing on incentives (56), not seeing people regularly (83) or having incentives to provide services themselves (56). Participants may be hampered to engage by their circumstances (56,75,76,81,83,88). Lastly, a small recruitment pool (75) and general difficulty to recruit (51,53,71,75,80,96) were reported. | Marketing (19,53), referral by healthcare professionals (18,19,72,76,79,82,94) who commonly see people (83), counsellors working within or close to antenatal clinics (87), using technology (86), a culturally fit and trusted recruiter and strategy (56,75,94), attractive rewards (85), more efforts (53,94) and variety of approaches (e.g. texts, calls, E-mail) (75), and self-enrolment options (93). | "Women would say yes when asked to join the study by a midwife but then not respond to [Research Assistant] contact attempts. Recruiting pregnant women from health professionals may not be the most effective strategy and other forms of recruitment need to be explored." (75) |
| Retention | Personal circumstances (19,49,94,96,52,56,71,76,81,82,88,90), loss of motivation after missing a reward (85), or participation being demanding (80,81), stressful (85), not or negatively promoted (75,81). | Frequent engagement (48,72,85,96) using technology (81), increasing understanding of underlying reasons (71), a personalised (51) and confidential approach (76), and offering incentives (19,76). | "It is important to maintain communication with the women who lapse and encourage them to re-engage." (96) |
| Involving stakeholders | Not involving the target population (56,68). | Involve stakeholders trusted by the community (19,56), policy makers or potential funders (56,68,94), or providers/referrers (56,76,87,90,94). | "Lack of involvement of the target population during development and implementation of the study (…) are limitations." (68) "Create a network of support. Go out and talk to doctors and other agencies and potential referral sources about the program." (64) |

## Supplementary Figure 1

**Overview of barriers and facilitators to the implementation of interventions with financial incentives.** BV: Biochemical Validation.
